# Combined petrosal approach for resection of recurrent brainstem cavernous malformation: operative video and technical nuances

**DOI:** 10.3171/2019.7.FocusVid.19229

**Published:** 2019-07-01

**Authors:** James K. Liu, Vincent N. Dodson

**Affiliations:** Department of Neurological Surgery, Center for Cerebrovascular and Skull Base Surgery, Neurological Institute of New Jersey, Rutgers University, New Jersey Medical School, RWJ Barnabas Health Newark, New Jersey

**Keywords:** combined petrosal, cavernous malformation, retrolabyrinthine, Kawase, petrosectomy, video

## Abstract

Brainstem cavernous malformations are formidable lesions because of their eloquent location and propensity for bleeding resulting in neurological impairment. The surgical management can be challenging due to their deep location around critical neurovascular structures. In this operative video manuscript, the authors demonstrate resection of a large recurrent pontine cavernous malformation with an exophytic component in the cerebellopontine angle via a combined petrosal approach. Both anterior and posterior (retrolabyrinthine) petrosectomies were performed to allow multi-corridor access to the lesion. Due to excessive scar formation from prior surgeries, sharp dissection was paramount to create dissection planes around the lesion. This video atlas demonstrates the operative technique and surgical nuances of the skull base approach, safe resection of the malformation through the operative corridor, gentle handling of the neurovascular structures and a multi-layered reconstruction technique to prevent cerebrospinal fluid leakage. The use of endoscopic-assisted microsurgery of the brainstem is also demonstrated. A gross total resection was achieved, and the patient improved neurologically. In summary, the combined petrosal approach with endoscopic assistance is an important strategy in the armamentarium for the surgical management of brainstem cavernous malformations.

The video can be found here: https://youtu.be/oAETW6tVc_Y.

## Transcript

This is Dr. James Liu. I’ll be demonstrating a combined petrosal approach for resection of a recurrent brainstem cavernous malformation.

### 0:30 Patient History

The patient is a 23-year-old male who had two prior surgeries for a hemorrhagic brainstem cavernous malformation. The patient was initially operated on at an outside hospital with a left retrosigmoid approach 3 years ago. The patient re-hemorrhaged due to a recurrence and was operated on by another surgeon using an endoscopic transclival approach 1 year later. He now presents to our center with worsening left-sided headaches, decreased sensation in the left V1 and V2 distribution, diplopia from a left sixth nerve palsy, a very dense left seventh nerve palsy, with decreased hearing on the left side, and worsening right-sided weakness.

### 1:16 Preoperative Imaging

T2 MRI demonstrates a recurrent cavernous malformation in the pons. Note that the cavernoma presents itself to the anterolateral aspect of the brainstem at the level of the trigeminal nerve. The cavernoma also has an exophytic component extending into the CP angle, down to the level of the medulla. Post-gadolinium T1 images show an enhancing deep venous anomaly, coursing through the middle of the pons in a sagittal direction.

### 1:48 Patient Positioning and Skin Incision

We therefore chose a combined petrosal approach, comprised of an anterior and posterior retrolabyrinthine petrosectomy. This allows for multi-corridor access via the Kawase’s approach and the presigmoid approach to the brainstem (Kawase et al. 1991, Cho and Al-Mefty 2002, Asaoka and Terasaka 2014, Arnaout and Al-Mefty 2017). Hearing can be preserved with this petrosal variant.

The patient is positioned supine with the head turned to the right side approximately 60 degrees. The right shoulder is elevated with a cushion to facilitate head turning. Incisions for an abdominal fat graft and fascia lata are prepared. Intraoperative navigation and lumbar drainage as well as facial nerve monitoring and brainstem auditory evoked responses are used.

We incorporated the patient’s previous S-shaped retrosigmoid incision and extended it anteriorly behind the hairline. The galeocutaneous flap is elevated, and the temporalis muscle is reflected anteriorly to expose the mastoid and the temporal bone flap.

A vascularized pedicled pericranial flap is raised and pedicled posteriorly for later use during reconstruction.

### 3:02 Extradural Dissection

After elevating the temporal craniotomy, extradural dissection is performed by elevating the temporal dura off the middle fossa floor. The GSPN is dissected from the dura in a posterior to anterior fashion, using sharp dissection. The middle meningeal artery is coagulated and divided at the foramen spinosum to allow further dural peeling off of V3. Bleeding from the posterior cavernous sinus is controlled with Surgiflo.

### 3:30 Anterior and Posterior Petrosectomy

The anterior petrosectomy is performed with a high-speed drill, medial to the GSPN, posterior to V3, and anterior to the arcuate eminence. Care is taken to skeletonize the horizontal petrous ICA. The posterior petrosectomy begins with a retrolabyrinthine mastoidectomy, identifying the lateral semicircular canal and the mastoid antrum. The posterior canal is skeletonized followed by the superior canal and the subarcuate fossa. We then skeletonized the sigmoid sinus and the presigmoid dura and sinodural angle. It is important to drill the bone behind the posterior canal to maximize the presigmoid corridor. This variation of the petrosal approach allows for possible hearing preservation. Once the superior canal is exposed, it is easier to maximize drilling of the postmeatal triangle. The bone is elevated off the IAC dura.

### 4:47 Dural Incision

Once the drilling of Kawase’s triangle is complete, the dura is opened along the temporal base. It is important to visualize the tentorial incisura subtemporally. The dura is then incised toward the lateral margin of the tentorium. The posterior fossa dura is also opened to meet the superior petrosal sinus. The sinus is then divided which can be controlled with bipolar cautery. This cut is continued towards the tentorial incisura, while identifying and protecting the fourth nerve. Once the cuts are complete, exposure to the brainstem, above and below the fifth nerve is achieved. The presigmoid dural incision is performed and a transtentorial cut is made in a similar fashion. However, there were dense adhesions in this region from prior surgery, so we focused our approach through the middle fossa Kawase’s corridor.

### 5:59 Resection of Cavernous Malformation

The exophytic component of the cavernoma was visualized below the fifth nerve. A plane of dissection was created with fine-bayonetted forceps. The cavernoma was removed in a piecemeal fashion with its associated hematoma. The lower portion of the cavernoma was removed by using gentle traction with the dominant hand and suction dissection with the nondominant hand. This technique allowed us to deliver the most inferior pole of the cavernoma that extended to the medulla. After clearing the inferior part of the brainstem, we turned our attention towards the upper pons, centered around the fifth nerve. There was hemosiderin-stained tissue above and below the fifth nerve, which correlated with the upper pole of the cavernoma on image guidance. We identified the cavernoma through a corticectomy below and then above the fifth nerve. Fine-bayonetted forceps are used to create a plane between the cavernoma and the surrounding gliotic tissue. We enlarged our corticectomy by combining the two corridors to better facilitate safer removal of the cavernoma. Again, piecemeal removal was performed by using gentle traction with suction dissection. Because the patient had prior surgeries, various portions of the lesion were densely adherent, requiring sharp dissection with microscissors to release the cavernoma. The deep venous anomaly was finally identified, coursing through the middle of the pons. There was some residual cavernoma adherent to the DVA, which was carefully dissected off, using sharp dissection with microscissors. It is critical to preserve the DVA in order to avoid venous infarction.

### 8:56 Endoscopic Inspection of Resection Cavity

The DVA was re-inspected and now appeared clear of any malformation. A 30-degree endoscope is used to look into the CP angle through Kawase’s triangle. The prepontine arachnoid is visualized. The resection cavity is carefully inspected, and the deep venous anomaly is visualized. Careful inspection of the resection cavity did not reveal any residual malformation.

### 9:27 Hemostasis and Closure

Hemostasis is achieved with Surgicel. The fourth and fifth nerves were visually intact, as were the seventh and eighth nerves on neuromonitoring. The mastoid antrum is occluded with bone wax to prevent a CSF fistula to the middle ear. The remaining air cells are sealed off with HydroSet cement. Fat graft was placed into the petrous apex defect and the dura was closed with a DuraGen inlay followed by an autologous fascia lata graft onlay that was tacked down with sutures. Surgicel was placed over the graft followed by Evicel fibrin glue. We then rotate the vascularized pedicled pericranial flap into the field and secure it with Surgicel. The bone flap is replaced, and the mastoid defect is repaired with a Medpor titan implant. Multi-layered wound closure is performed in the standard fashion. The double petrosectomy defects are visualized on the temporal bone CT with preservation of the otic capsule.

### 10:39 Postoperative Course

Postoperative MRI did not show any residual cavernous malformation. The patient remained neurologically stable in the immediate postoperative period without any new deficits. At 2-months follow-up, he had significant improvement of his sixth and seventh nerve palsies, which were completely resolved at the 5-month follow-up visit. At one-year follow-up, he had a normal cranial nerve exam with significant improvement of his strength on the right side. Hearing was also improved and remained intact. MRI at one-year follow-up showed no recurrence with an intact deep venous anomaly coursing through the pons.

### 11:19 Discussion

We typically would use a Kawase’s approach for brainstem cavernous malformations that present to or near the surface of the anterolateral pons, between cranial nerves V and VII (Giliberto et al. 2010). Lesions that extend superior to cranial nerve V can also be accessed by this approach, as demonstrated in this video. If, however, the lesion extends below the IAC and the seventh and eighth nerves, we would add a posterior petrosectomy approach to get lower on the clivus and petroclival region (Gross et al. 2012). Based on the preoperative MRI, the cavernoma extended from the upper pons down to the upper medulla, below the IAC. Therefore, we chose a combined petrosal approach in this case to get maximal exposure to the cavernous malformation. However, there were significant scar tissue and adhesions in the presigmoid window due to a prior retrosigmoid surgery, so we therefore primarily worked from the Kawase’s approach, which actually proved very favorable, especially with endoscopic assistance. We were able to get much lower than expected down on the clivus due to the cavernoma having a significant exophytic component as demonstrated on the preoperative MRI. Although the presigmoid corridor was not used, the addition of the posterior petrosectomy also allowed for more surgical freedom and less temporal lobe retraction due to a wider working corridor.

### 12:45 Conclusion

In summary, the combined petrosal approach with endoscopic assistance is an important strategy in the armamentarium for the surgical management of brainstem cavernous malformations.
